# Incontinentia pigmenti presenting as hypodontia in a 3-year-old girl: a case report

**DOI:** 10.1186/1752-1947-3-116

**Published:** 2009-11-10

**Authors:** Dárcio Kitakawa, Patrícia Campos Fontes, Fernando Augusto Cintra Magalhães, Janete Dias Almeida, Luiz Antonio Guimarães Cabral

**Affiliations:** 1 Department of Bioscience and Oral Diagnosis, São José dos Campos Dental School, São Paulo State University-UNESP, São José dos Campos-SP, Brazil

## Abstract

**Introduction:**

Incontinentia pigmenti or Bloch-Sulzberger syndrome is a rare X-linked dominant disease that mainly affects the skin, eyes, hair, central nervous system and teeth. The disease is predominant among women. Although dermatologic manifestations are among the most important aspects for the diagnosis of the syndrome, they are less damaging to the patient and do not require treatment. However, oral involvement characterized by hypodontia of deciduous and permanent teeth is important for the diagnosis and treatment of the patient.

**Case presentation:**

We report the case of a 3-year-old girl with ophthalmologic and neurologic disturbances, cutaneous manifestations and hypodontia. Since the patient did not present more damaging manifestations such as neurologic and/or ophthalmologic problems, her most severe complications were related to dental anomalies. The importance of integrated dental treatment, which combines pediatric dentistry, orthodontics and conventional prosthesis, is emphasized.

**Conclusion:**

Hypodontia is a frequent finding in incontinentia pigmenti, and dentists should be aware of this condition in order to help with the diagnosis.

## Introduction

Incontinentia pigmenti (IP) or Bloch-Sulzberger syndrome is a rare X-linked dominant genodermatosis which mainly affects women. The disease is systemic and involves tissues of ectodermic and mesodermic origin including cutaneous tissue, teeth, eyes and the central nervous system (CNS), amongst other organs [1-4]. The name incontinentia pigmenti is related to the histological characteristics of the disease, that is, melanin incontinence by melanocytes in the basal epidermal layer and its presence in the superficial dermis in the final stage of the disease [5].

IP is a single-gene disorder caused by mutations in the NEMO/IKK-γ gene. The function of NEMO, a 23-kb gene consisting of 10 exons, is to permit cells to respond to external signals such as growth factors [5]. This gene encodes a protein that regulates the function of various chemokines, cytokines and adhesion molecules, and is essential for protection against tumour necrosis factor-induced apoptosis [1,5,6].

The inheritance of a mutant copy of this X-linked gene is generally lethal in antenatal males. Although there are many case reports of men with IP submitted to mutation analysis, none of these patients has been shown to carry the common NEMO deletion. Several studies have demonstrated the presence of hypomorphic NEMO alleles, a finding suggesting that less severe NEMO mutations permit the survival of affected men [6,7]. There are also several reports of men with IP who present the 47, XXY karyotype (Klinefelter syndrome) [8,9].

Cutaneous manifestations of IP are classically divided into the following four stages, although not all stages may be present in some cases [2]: stage 1 - erythema, vesicles and blisters appearing in a typically linear pattern; stage 2 - papulae, verrucous lesions and hyperkeratosis; stage 3 - hyperpigmentation; stage 4 - hypopigmentation and cutaneous atrophy. In all of these stages, the cutaneous lesions tend to follow the Lines of Blaschko [2,10].

Following dermatologic alterations, dental manifestations are the most frequent, which are observed in 80% of patients and usually affect both dentitions. The most frequent alteration is hypodontia (up to 43% of patients), followed by pegged or conically crowned teeth (30% of patients). These manifestations are important because they persist throughout the patient's life, thus requiring an adequate dental treatment plan from the time of diagnosis of the disease to oral rehabilitation by a multidisciplinary team [11,12].

We report the case of a 3-year-old girl in which a dentist contributed to the diagnosis of IP.

## Case presentation

**Figure 1:**
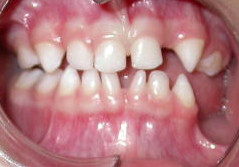
**Intraoral view showing the absence of some deciduous teeth (62, 74 and 75)**. Note the presence of conoid teeth (71 and 82).

**Figure 2:**
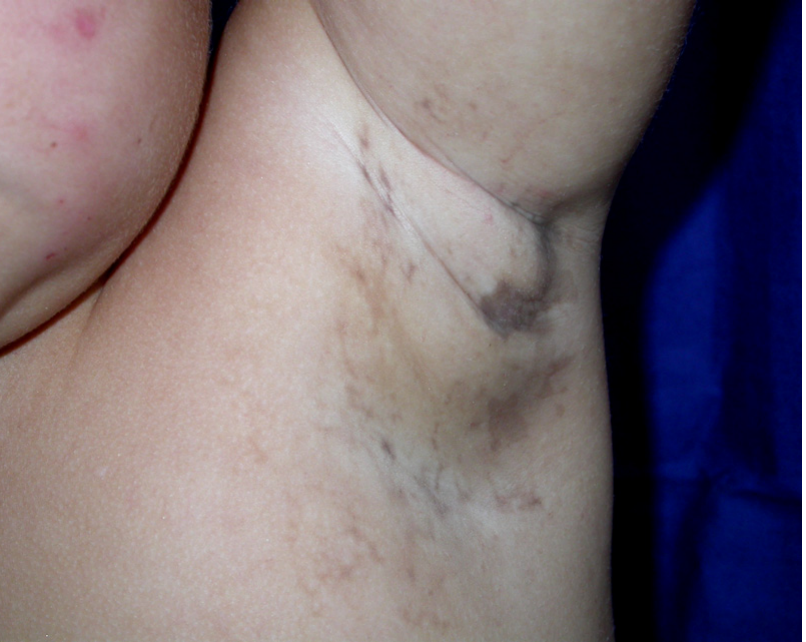
**Region of the left axilla**. Note the presence of characteristic brown-grayish maculae following the Lines of Blaschko.

A 3-year-old Caucasian girl accompanied by her mother was referred by her pediatric dentist to the outpatient stomatology clinic at the São José dos Campos Dental School, UNESP, because of the lack of eruption of some teeth and marked atrophy of the left posterior inferior alveolar border (Figure 1). During investigation of her medical, dental and familial history, the mother reported the presence of maculae in various regions of the child's body. This was confirmed by physical examination, with the observation of bilateral, slate-brown maculae in the axillary region (Figure 2), on the back, and in the hypochondrium and groin. With respect to clinical history, the mother reported that her daughter had suffered some episodes of seizures when she was approximately one month old, and that at that time small blisters had appeared that followed the same trajectory as the current maculae. During that time, the reported blisters were diagnosed as herpes zoster and treated with topical acyclovir, but the outcome was unsatisfactory. After a period of approximately one month, the mother noted remission of the bullous lesions; however, maculae started to develop in these regions and persisted until the time of the visit.

**Figure 3:**
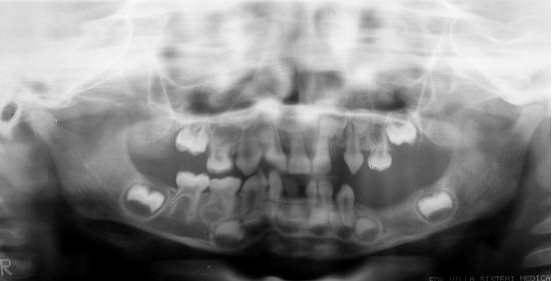
**Orthopantomographic radiograph confirming the absence of deciduous (62, 74 and 75) and permanent teeth, mainly in the left mandibular area, and characterizing radicular and coronal changes**.

The physical and intellectual development of the girl was considered to be normal by pediatricians despite the presence of discrete strabismus. After extra- and intraoral examination, a panoramic X-ray was requested to evaluate tooth germ development (Figure 3) and to determine the reason for the lack of some teeth in the region of the third quadrant and the presence of conoid teeth 71 and 82. The X-ray revealed the absence of some deciduous and permanent tooth germs in the third quadrant (74, 75, 34, 35), as well as the lack of tooth 62 and of various permanent tooth germs at other sites. The mother was asked about the existence of family members with similar signs, which she denied. Hematologic tests showed no alterations.

Based on the clinical history and clinical and radiographic findings, the final diagnosis was IP [9]. At present, the girl is under complete medical follow-up, mainly taking into account alterations in the skin, eyes, CNS and mouth.

## Discussion

The clinical manifestations of IP vary widely even in the same family, ranging from subtle cutaneous and dental changes to severe and incapacitating ophthalmologic and neurologic manifestations [11]. The latter are the most serious clinical manifestations of IP. In our case, the patient showed no severe ophthalmologic manifestations except for strabismus which, although not a retinal-related symptom, is the most frequent ophthalmologic alteration, ranging in reports from 18% to more than 33% of patients [1,2,11]. More severe retinal-related lesions generally become evident after the neonatal period and during the first year of life. The prognosis for children who do not develop these manifestations is good, but continuous ophthalmologic follow-up is recommended. With respect to neurologic problems, the patient had episodes of seizures when she was approximately one month old, but had no further episodes thereafter. Seizures are the most frequent CNS-related manifestations of IP [2,9,11]. No other manifestations resulting from neurologic problems were detected by the neurologists.

The four stages of IP are not always present, with stages 1 (inflammatory or vesicular) and 3 (hyperpigmented) being more common than stages 2 (papular or verrucous) and 4 (hypopigmented or atrophic) [1,2,6]. In our case, the mother observed only stages 1 and 3. After rupture of the vesicles, the mother noted that these regions became darker than neighboring areas during healing. Clinical examination revealed hyperpigmented regions of a slate-brown color, which are part of the current clinical presentation of the patient, with these lesions characterizing stage 3 of IP. Since the patient is only 3 years old, she may possibly still develop stage 4 which can even include the disappearance of all lesions. Therefore, although dermatologic manifestations are one of the most important aspects for the diagnosis of the syndrome, they result in less damage to the patient and do not require treatment.

Since the girl did not present the more damaging neurologic and/or ophthalmologic manifestations, her most severe complications were related to the stomatognathic system [1,3,12-14]. Dental abnormalities have been found in 36 of 45 cases [6]. Radiographic examination revealed the absence of various permanent tooth germs (teeth 34 and 35), which in the future will lead to problems in masticatory and occlusal function if not treated adequately, and probably psychosocial problems due to a compromised esthetic appearance. The patient also presented hypodontia of deciduous teeth (62, 74 and 75) and conoid teeth (71 and 82), aspects that concur with the clinical findings of the syndrome. For these reasons, the girl was referred for integrated dental treatment combining pediatric dentistry, orthodontics and conventional prosthesis [13,14], with less concern regarding the disease but more emphasis given on the treatment of hypodontia, as well as on psychological support for the girl and her parents.

## Conclusion

Hypodontia is a frequent finding in incontinentia pigmenti, and dentists should be aware of this condition in order to help with the diagnosis and in the guidance of patients and their families.

## Consent

Written informed consent was obtained from the patient's parents for publication of this case report and accompanying images. A copy of the written consent is available for review by the Editor-in-Chief of this journal.

## Competing interests

The authors declare that they have no competing interests.

## Authors' contributions

All the authors analyzed and interpreted the patient data regarding the clinical and radiographic aspects. DK, PCF and FACM investigated the patient's medical, dental and family history. LAGC and JDA were major contributors in writing the manuscript. All the authors have read and approved the final manuscript.
